# A Context-Aware Mobile User Behavior-Based Neighbor Finding Approach for Preference Profile Construction †

**DOI:** 10.3390/s16020143

**Published:** 2016-01-23

**Authors:** Qian Gao, Deqian Fu, Xiangjun Dong

**Affiliations:** 1 School of Information, Qilu University of Technology, #3501 Daxue Road, Changqing District, Jinan 250353, China; dxj@qlu.edu.cn; 2 School of Informatics, Linyi University, Shuangling Rd., Lanshan, Linyi 276005, China; fudeqian@lyu.edu.cn

**Keywords:** multi-agent, context, trust degree, interest similarity degree, time attenuation

## Abstract

In this paper, a new approach is adopted to update the user preference profile by seeking users with similar interests based on the context obtainable for a mobile network instead of from desktop networks. The trust degree between mobile users is calculated by analyzing their behavior based on the context, and then the approximate neighbors are chosen by combining the similarity of the mobile user preference and the trust degree. The approach first considers the communication behaviors between mobile users, the mobile network services they use as well as the corresponding context information. Then a similarity degree of the preference between users is calculated with the evaluation score of a certain mobile web service provided by a mobile user. Finally, based on the time attenuation function, the users with similar preference are found, through which we can dynamically update the target user’s preference profile. Experiments are then conducted to test the effect of the context on the credibility among mobile users, the effect of time decay factors and trust degree thresholds. Simulation shows that the proposed approach outperforms two other methods in terms of Recall Ratio, Precision Ratio and Mean Absolute Error, because neither of them consider the context mobile information.

## 1. Introduction

### Main Idea of This Paper

The Internet has become the main source for people to obtain and exchange information, but it has also brought the problem of information overload. To solve this problem, personalized information recommendations and prediction technology have been employed to provide personalized search results for each user. The core issue of the technique is to build users’ preference profiles to help users identify the relevant information among the huge volume of available information.

For this purpose, we are researching on how to provide the end users with accurate and useful information which can meet their needs in accordance with their interests and track the changes by recording the user context and personalized applications. In order to facilitate the monitoring of human activities and the integration between humans and agents, the research proposes a novel Personalized Ontology Profile (POP) construction approach by comprehensively considering the domain ontology, knowledge structure, and the document information stored on all of the smart devices of the same user. Then, based on POP a user preference profile is constructed to track the users’ long-term behaviors and short-term behaviors with two kinds of user interest tracking strategies (Sliding-Time-Window Update Strategy for short term preferences, and Time-Based-Forgetting Function Update Strategy for long term preferences). Moreover, the contextual information (interactive historical information and user information related with the retrieval) stored in all of the smart devices owned by the same user (such as documents, E-mail, pictures and so on) is also adopted to more accurately grasp the relevance of different documents.

The users of the mobile network are influenced more by the context compared with those of the traditional Internet. In recent years, along with the development and application of 4G networks, ubiquitous computing, the Internet of Things technology, cloud computing, and the continuous improvement of the software and hardware capabilities of mobile terminals, abundant real time mobile users’ behavior data can be obtained from the mobile network, including information about the mobile users (such as gender, name, career and income), the location information (such as the position, the surrounding people and the weather) and the interactive behaviors of the mobile users. The mobile network has the characteristics of “position”, “real time”, “dynamic interaction” and “identity binding”, and more real social relations can be obtained through mobile network than through the Internet, which makes it inadequate to glean users’ preference merely based on the contextual information from the Internet, and the context of the mobile users needs to be included to build and update users’ preference profiles so as to continuously capture the changes in the users’ interests.

However, there are still some problems for obtaining user preferences in the current context-based dynamic mobile methods. Firstly, the calculation approaches cannot achieve a high trust degree accuracy between different mobile users. Secondly, the introduction of the context brings about the problem of sparsity, reducing the accuracy of the user preference predicted by the context of mobile users. Thirdly, some mobile users’ preferences change frequently, which cannot be captured instantly by the traditional offline methods. Therefore a novel neighbor searching approach based on mobile user behavior is proposed to solve the above problems.

This paper aims at finding the mobile user’s neighbors with similar interests by comprehensively considering the credibility and preference similarity of different mobile users so as to provide the foundation to update the target user’s preference profile. In addition, since the mobile phone input/output capability is limited, and the mobile users have real-time access to information, higher requests are put forward on the prediction precision of mobile user preference in the mobile network. The traditional prediction method of user preference is not well suited for personalized mobile network service systems. We therefore focus on how to introduce into the mobile user’s preference similarity calculation the context-based trust degree, preference similarity and the time attenuation trend, on the basis of which we can update the target user’s preference profile according to the dynamic neighbor user preference profile that is built based on users’ daily behavior on desktop and mobile networks.

The first innovation of this paper is that the users with similar interests are sought based on the context from mobile network services instead of from desktop networks. The second innovation is that it uses the trust degree and the similarity degree of mobile users with different mobile network services to search for neighboring users with similar interests. We first calculate the trust degree between mobile users by analyzing their behavior based on the context, and then we choose the approximate neighbors by combining the similarity of the mobile user preferences and the trust degree. The proposed method also takes into consideration the communication behaviors (speech, duration, speech frequency, message frequency, along duration, along frequency) between mobile users, mobile network services used by mobile users as well as the corresponding context information (time, position). We first calculate the direct trust degree based on the mobile users’ behavior and the weight of the corresponding context; then we propose an indirect trust degree calculation method according to the propagation distance based on the theory of the six degrees of separation. Second, we further use the evaluation score of a certain mobile web service given by a mobile user to calculate the preference similarity degree between different users. Third, based on the time attenuation function, we search for users with similar preferences to find the approximate neighbors.

The rest of this paper is organized as follows: Section 2 combs the studies related to the present research; Section 3 describes the framework and the realization of the proposed approach. The datasets and steps used in the simulation are presented in Section 4, and then provides the result and the discussion of the simulation. The conclusions are drawn in Section 5.

## 2. State-of-the-Art

At present, it is difficult to provide end users with accurate and useful information, because users’ interests change with time and it would require recording the user context and personalized applications to track the changes so as to meet their needs. To solve this problem, some researchers have utilized the user profile approach to offer each user personalized search results. Armin, who has presented three important approaches toward a Collaborative Information Retrieval System, confessed that: “Personalization means that different users may have different preferences on relevant documents, because of long-term interests; context means that different users may have different preferences on relevant documents, because of short-term interests [1]”. Therefore, if we can monitor users’ real-time behavior and preferences, based on this we can accurately grasp users' searching intentions, and as such, the retrieval system can understand users’ meaning more clearly, and send back more relevant documents according to their needs.

In order to build a user profile, some source of information about the user must be collected through direct user intervention, or implicitly, through agents that monitor user activity. Although profiles are typically built merely from topics of interest of the user, some projects have pursued the method to include information about non-relevant topics in the profile [2,3]. In these approaches, the system is able to use both kinds of topics to identify the relevant documents and meanwhile discard non-relevant ones. Hence, in general, a good user preference profile should comprise the results of lexical analysis, the input query, the documents clicked by the user, the previous queries of the user, and some weight values. However, a user preference profile involving incorrect user preferences may cause trouble to users. Incorrect user preferences are generally obtained by the static profile approach. In this static profile approach, preferences or weight values do not change over time once the user preference profile is created. In contrast, dynamic profiles can be modified or augmented and can be classified into short-term and long-term interests with time taken iton consideration [4,5]. Short-term profiles represent users’ current interests, whereas long-term profiles indicate interests that are not subject to frequent changes over time. To solve this problem, some researchers have explored methods to dynamically update user preferences by taking the knowledge structures into consideration. 

**Figure 1:**
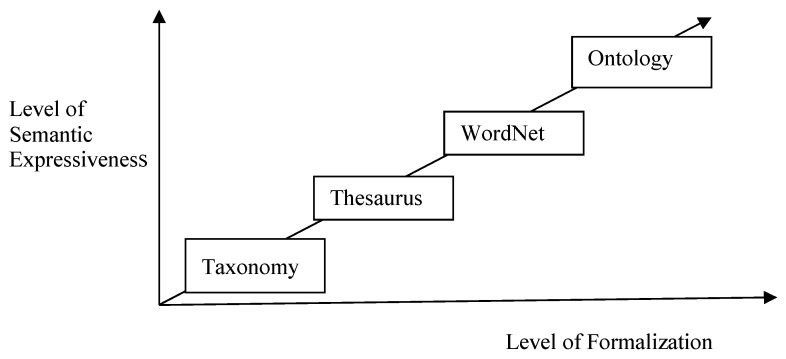
Four types of knowledge structures.

There exist four kinds of knowledge structures varying in the levels of formalization and semantic expressiveness, as shown in Figure 1. For the depicted knowledge structures it can be stated that the higher their level of formalization is, the better their semantic expressiveness is.

WordNet is a special type of thesaurus that constitutes a widely used source for the implementation of query expansion mechanisms. As stated by Fellbaum [6], “WordNet is neither a traditional dictionary nor a thesaurus but combines features of both types of lexical resources”. First, WordNet interlinks not just word forms or strings of letters, but specific senses of words. As a result, words that are found in close proximity to one another in the network are semantically disambiguated. Second, WordNet labels the semantic relations among words, whereas the groupings of words in a thesaurus do not follow any explicit pattern other than meaning similarity. The main relation among words in WordNet is synonymy, as the relationship between the words shut and close or car and automobile. Additionally, a synset contains a brief definition (“gloss”) and, in most cases, one or more short sentences illustrate the use of the synset members. Word forms with several distinct meanings are represented in many distinct synsets (Word Net).

Usually, the same concept has more than one expression; therefore, semantic network profiles in which the nodes represent concepts, rather than individual words, are likely to be more accurate. The SiteIF [7] system builds this type of semantic network-based profile from implicit user feedback. Essentially, the nodes are created by extracting concepts from a large, pre-existing collection of concepts, WordNet [8]. The keywords are mapped into the concepts with WordNet. Every node and every arc has a weight that represents the user’s level of interest. The weights in the network are periodically reconsidered and possibly lowered, depending on the last updating time.

An ontology offers a superior level of expressiveness to Word Net, in which all imaginable semantic relations can be defined between all kinds of objects. The concept of ontology was developed in Artificial Intelligence to facilitate knowledge sharing and reuse. In computer science, an ontology typically consists of a set of classes and the definition of relationships (relations) that exist between objects of these classes. These objects are referred to as instances which are sometimes subsumed under the term ontology. Different fields need to build different domain ontologies. Computers exchange information between different fields through the understanding of the ontology. Each document on the semantic web is an ontology, and a big document can be divided into several small ontologies.

Ontology has been proven to be an effective means for modeling digital collections and user profiling. In the field of ontology design, efforts have been made by several research groups to facilitate the ontology engineering process, with both manual and semi-automatic methods. A. Maedche *et al*. [9] proposed a framework with the semiautomatic method which incorporates several information extraction and learning approaches, in order to face the discovery of relevant classes, their organization in a taxonomy and the non-taxonomic relationships between classes. Gauch *et al*. [10] also proposed a system that adapted information navigation based on a user profile structured as a weighted concept hierarchy. The above studies prove ontology to be an effective tool, because it may present an overview of the domain related to a specific area of interests and be used for user preference classification.

Furthermore, as mentioned in Section 1, most researchers merely combined the context information obtained from the traditional internet and domain ontology to build personalized user profiles, but they failed to consider the effect of the mobile network itself. To solve this problem, we analyze the context of the mobile network users, based on which, we construct and update the user preference profile, so as to further enhance the accuracy of the prediction of user preference.

Nowadays, trust degrees can be obtained in two ways. They can be obtained explicitly through questionnaires, scoring, the behavior of users’ friends in the Social Networking Services, *etc*. Even though the situation is getting better in parallel with the constant improvement of smartphone functions, due to the input and output capability limitations of mobile phones, the explicit information about trust degree is still far from enough. The trust degree can also be acquired implicitly through the analysis of the behaviors of the mobile users, but currently it mainly includes some simple analysis such as the speech duration, speech frequency and message frequency. Because of the improving performance of smart phones, the real time behaviors of mobile users can be obtained in this way, and as such, most researchers adopt this method to acquire the trust degree. However, few researchers consider the context, the social influence of mobile users, as well as the influence of the preference similarity degree on trust degree between mobile users. The most classical and widely used method to forecast the user preference is by using some type of collaborative filtering algorithm. The core concept is to search for the similar neighbors of the target user by calculating the similarity among different users, and then forecast the target users’ interests according to the preference of neighbors [11]. However, along with the expansion of the data scale, the problem of sparsity gradually looms, leading to the declination of the efficiency and the accuracy of the algorithm. Some researchers try to solve this problem by integrating the trust relationship between users into the traditional collaborative filtering algorithm.

The trust degree between mobile users is not only connected with the interbehavior of different mobile users, but also connected with the context, the social influence of mobile users and the influence of the preference similarity degree between them. Quercia [12] monitored the behavior of mobile users by the message, the speech communication and the information obtained from Bluetooth, and recommended the neighbors with similar interests according to the obtained behaviors. Eagle [13] deduced the friend relationship between different mobile users by analyzing the communication behavior, time, location and surrounding people, but she did not provide specific calculating method of the trust degree. By calculating the similarity degree of different mobile users in real data, Woerndl [14] found that users with closely connected relationships have higher preference similarities. 

Wang [15] proposed a user-service-context three-dimensional collaborative filtering model. With the user context information, she improved the accuracy and reliability of service selection, meanwhile avoiding the blindness and arbitrariness. However, when the influence of the context is taken into consideration, the original user-item scoring matrix is expanded into user-item-context three-dimensional matrix, which intensifies the sparsity problem. She proposed two approaches to solve the above problem. The first one is to reduce the dimensions of the context-based matrix. The other one first finds the approximate neighbors by merely considering the user-item matrix, and then predicts the preferences of the approximate neighbors by taking the context information into consideration. Furthermore, in order to effectively alleviate the problem of sparsity, trust degree matrix obtained explicitly is integrated with similarity preference matrix of the mobile user preference to select the target users’ approximate neighbors. Huang [16] proposed a collaborative filtering algorithm with the social network analysis method based on users’ social relationship mining, and by applying it to the mobile recommendation system, she effectively mitigated data sparsity. She further calculated the direct trust degree between mobile users according to their communication behavior, and accordingly, obtained the indirect trust degree between them so as to fill in the trust degree matrix. Huang [17] proposed a new trust model named Email Trust (EMT) constructed on the basis of the interactions among users via their daily emails, and provided a level of belief on the data transmitted by a user. In EMT, each user performs a trust checking procedure and processes a received trust checking request from his highly trusted email contacts or through a trusted checking proxy server maintained by a trusted third party. 

The preferences of some context-based mobile users change with time. To provide an accurate real time personalized service, user preferences based on the context need adaptive updating. The adaptive learning methods of user preference include explicit adaptive study and implicit adaptive study. The explicit adaptive study means that users update the preferences according to the feedback such as marking [18]. Implicit adaptive study updates preferences by monitoring and machine study [19]. Rao considered the influence of the context on user preference and digs user preference by analyzing users’ behavior connected with the context, but she failed to take changing preferences into consideration [20]. Xie aimed at solving the problem that personalized information service cannot be adjusted to the change of users’ demands, and analyzed the reasons of the change from the perspective of behavior motivation [21]. She judged implicitly the changing tendency and responded to it actively, but she did not consider the influence of the context on user preference, which compromised the accuracy of the service.

Furthermore, some researchers introduced the context information into the intelligent recommendation system, which can be used to analyze user preference behavior so as to improve the quality of prediction and recommendation. Location, time, sound, change of user preference over time and change created by mood swings all belong to the influence caused by context information. Different from the traditional Internet, the mobile communication network with the characteristic of mobility has access to the Internet whenever and wherever; therefore the effect of the context information on mobile user preference is more conspicuous.

## 3. The Context-Awareness Mobile User Preference Profile Construction Approach and Experiments 

### 3.1. The Framework and Implementation of the Proposed Method

The traditional user preference construction methods based on ontology normally classify the concepts according to the general domain ontology, which cannot indicate the difference between individuals. Therefore, in the prior research, we first combine the method of WordNet and an ontology to build an improved domain ontology tree, using WordNet to identify concepts based on the overlap of the local context of the analyzed word with every corresponding WordNet entry. Simultaneously, the ontology-based representation allows the system to use fixed-size document vectors, consisting of one component per base concept. Second, a Personalized Ontology Profile (POP) was constructed by considering the improved domain ontology tree, knowledge structure, and the document information stored on all of the smart devices that belong to the same user. Third, based on POP and context information, a preference profile is constructed which can comprehensively track the users’ long-term behaviors and short-term behaviors. Finally, we use fuzzy c-means clustering method to collect the user’s interest domain to determine whether the user’s documents of interest belong to the current interest-domain, based on which we can update the user profile, so as to continuously capture the changes of the users’ interests.

However, the previous research merely considers the context information obtained from the traditional Internet that fails to consider the effect of the mobile network. Along with the rapid development and application of tri-networks integration, ubiquitous computing and Internet technology, mobile networks expand the range of information services, which provides richer mobile network services than the traditional communication service. Meanwhile, due to the increasing popularity of smart mobile devices, sensors and radio frequency identification, information acquisition and push can occur at any time, in any place and by any way, which renders it possible to provide the mobile users with ubiquitous mobile network services. Compared with the traditional Internet, the acquisition of mobile user preferences is facing dynamic, diverse and ubiquitous mobile network environments, so the impact of context for mobile users is more obvious. Built on the above reasons, in this paper we introduce the context of the mobile network users into the construction and the updating of the user preference profile, so as to further enhance the accuracy of the prediction of user preferences.

**Figure 2:**
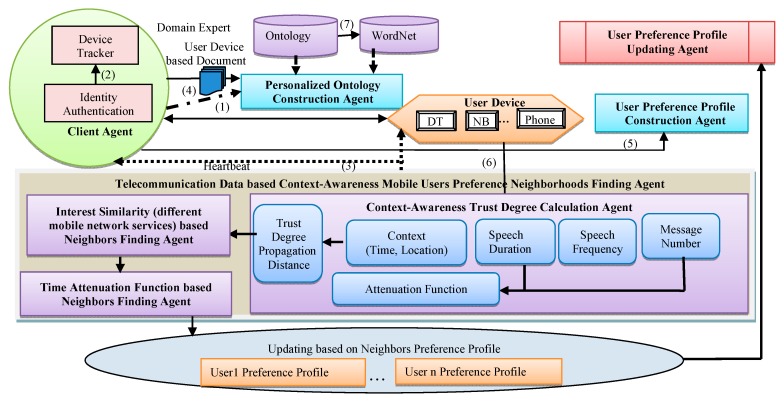
The framework of the proposed system.

This paper explores the approach to search for the neighbor users with similar interests by comprehensively considering the communication behavior, duration time, the mobile web services and the context information (time and position) of different mobile users. The neighbor user preference profile can be one of the influential factors that can dynamically update the target user’s preference profile. Thus, the approach proposed in this paper is an integrated part of the personalized information retrieval system based on context-awareness user preference profile, as shown in Figure 2.

First, the Client Agent proposed by Gao [22] is adopted to verify user identities through creating a union user account and to monitor whether a device is ready to implement a personalized user preference profile creation task. Second, a Personalized Ontology Construction Agent proposed by Gao [23] is used to create a personalized ontology profile, an extension of domain ontology which considers both the Knowledge Structure and the users’ behavior. Third, a three-level User Preference Profile Construction Agent proposed by Gao [23] is used to calculate and update users’ degree of interest by comprehensively considering users’ local behavior, browsing behavior, new input query, and pace times. 

Fourth, based on telecommunication data, a context-awareness mobile user preference neighborhood finding agent is used to identify the neighbors with similar interests by comprehensively considering the trust degree among different mobile users, interest similarity of mobile users with different mobile network services and time attenuation functions. Finally, the User Preference Profile Updating Agent is used to update the target user’s preference profile based on the neighbors’ preference profile.

### 3.2. Telecommunication Data-Based Context-Awareness Mobile User Preference Neighborhood Finding Agent

#### 3.2.1. Used Data Set

There is a large amount of users’ communication and interaction information in mobile networks, such as voice communication, text messages and other communication records and web browsing, software downloading, e-commerce, E-mail and other value-added services. The present research builds data set according to the information below:

(1) Collection of mobile web service contents used by users: (*U_i_*, *S_ij_*) indicates the network services *S_ij_* (*j* ∈ [1,m]) used by user *u_1_*, *u_2_*, …, *u_n_*.
(2) The evaluation score about the web service *S_ij_* graded by users: *S* (*S_1_* < *u_i_*, *S_i1_* >, *S_2_* < *u_i_*, *S_i2_* >, …, *S_t_* < *u_i_*, *S_it_* >) (*t* <= *m*). Here < *u_i_*, *S_ij_* > is the evaluation score about the web service *S_j_* given by user *u_i_*. The evaluation score of a certain web service is first mined by the feedback information and evaluation record of a user about different services that he/she used, and is then comprehensively amended by the usage condition about the related mobile services of a user.
(3) Trust degree set of mobile users: *TR_ij_* < *u_i_*, *u_j_* > indicates the trust degree of mobile users *u_i_* and *u_j_*. The trust degrees of different mobile users are obtained by analyzing communication behavior among different mobile users. The more frequently mobile users *u_i_* and *u_j_* contact with each other, the higher is the trust degree between them.

#### 3.2.2. The Overview of the Proposed Algorithm.

**Figure 3:**
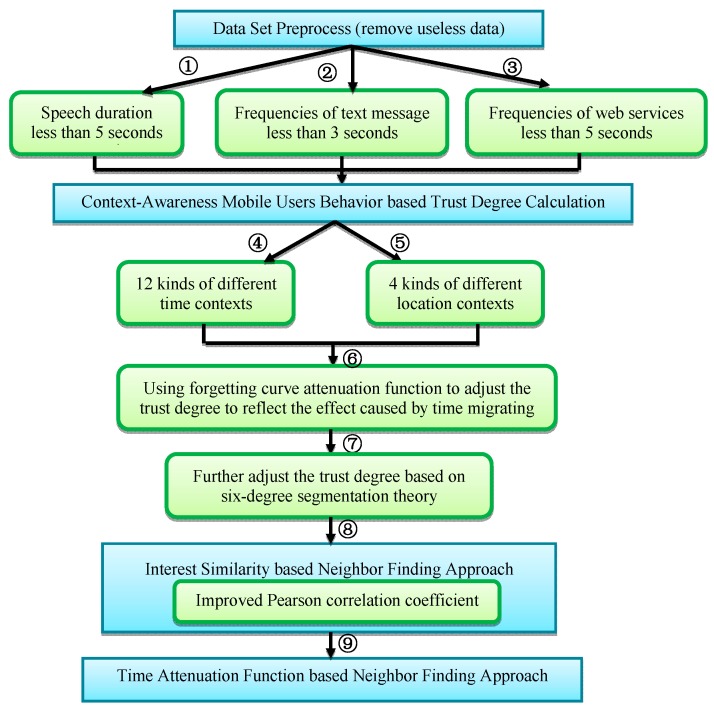
Overview of the proposed algorithm.

The overview of the proposed algorithm is shown in Figure 3.

Firstly, the irrelevant information may interrupt the analysis and the construction of the neighbor area of a certain mobile user, hence we preprocess the dataset before it flows into the processing scheme according to three circumstances so as to remove the intervening effects of the irrelevant information (shown as routes ①②③ in Figure 3). Secondly, it is important to include time, location, members around, and the corresponding behavior information among mobile users when calculating the trust degree between mobile users, and as such, different weights for various kinds of circumstance in different contents were set as the computation basis (shown as routes ④⑤ in Figure 3). Moreover, trust degree between mobile users will also change with time; therefore, an attenuation function called forgetting curve is used to adjust the trust degree based on different context (shown as route ⑥ in Figure 3). Furthermore, a method according to the six-degree segmentation theory is put forward to calculate trust propagation distance to acquire the more accurate context awareness trust degree (shown as route ⑦ in Figure 3). Thirdly, an improved Pearson correlation coefficient is adopted to calculate the preference similarity degree between different users with the evaluation score about a certain mobile web service provided by a mobile user so as to find the approximate neighbor (shown as route ⑧ in Figure 3). Finally, a logistic function is adopted to calculate the time attenuation based users’ preference weight by comprehensively considering the trust degree and similarity degree (shown as route ⑨ in Figure 3).

#### 3.2.3. Data Preprocessing

In order to avoid the intervening effect of the irrelevant information, when analyzing and building the neighbor area of a certain mobile user, the present research reprocesses the data set before calculating the trust degree and similarity score as shown in the following:

(1) Conversation information with the speech duration less than 5 s is deleted.
(2) The frequencies less than 3 are deleted as the noneffective text message conversation behavior.
(3) Record the information about web services used by the mobile users, and set the using frequency threshold as 5. The frequencies less than the threshold 5 are deleted as misoperations or disinterested network services.

#### 3.2.4. Context-Awareness Mobile Users Behavior Based Trust Degree Calculation

If mobile users have frequent communication behaviors, a direct trust relationship is considered to exist between them. Moreover, if mobile users have common trust neighbors instead of frequent communication behaviors, they have indirect trust relationship. High trust degree between users tends to influence user preference between each other, and as such, besides user preference similarity, the present research includes the trust degree between users as the second influential factor to determine the approximate neighbor collection of target users, and accordingly to update mobile user preference database.

When calculating the trust degree between mobile users, we consider time, location, members around, and the corresponding behavior information among mobile users. In this paper, the context of time and location are classified according to the rules of Equations (1) and (2):

(1) $$\{\begin{array}{c} \mathrm{Workday}\begin{array}{ccc} & & 0 \end{array}\\ \mathrm{Weekend}\begin{array}{ccc} & & 1 \end{array} \end{array}\begin{array}{cc} & \mathrm{and}\{\begin{array}{c} 00:00-08:30\begin{array}{cc} & 0 \end{array}\\ 08:30-12:00\begin{array}{cc} & 1 \end{array}\\ 12:00-13:30\begin{array}{cc} & 2 \end{array}\\ 13:30-17:30\begin{array}{cc} & 1 \end{array}\\ 17:30-21:30\begin{array}{cc} & 3 \end{array}\\ 21:30-24:00\begin{array}{cc} & 2 \end{array} \end{array} \end{array}$$

(2) $$\{\begin{array}{c} \mathrm{hom}e\begin{array}{cc} & 0 \end{array}\\ \mathrm{party}\begin{array}{cc} & \begin{array}{cc} & 1 \end{array} \end{array}\\ \mathrm{workplace}\begin{array}{cc} & 2 \end{array}\\ \mathrm{other}\begin{array}{ccc} & & 3 \end{array} \end{array}$$

In the following text, several terms are used with the meanings defined in Table 1.

**Table 1:** The index of terms used in this paper.

Terms	Meaning
speech duration	The time users spent on voice calls
speech frequency	The number of the voice calls between two users
message frequency	The number of the text messages between two users
Along duration	The total time of two uses when they stay in a short distance (such as in the same office) which is within the range of Bluetooth detection.
Along frequency	The total number of two uses when they stay in a short distance (such as in the same office) which is within the range of Bluetooth detection.

The more communication two users have, the higher is the trust between them. Then the trust degree between users is calculated based on the user speech duration, speech frequency and message frequency, as shown in Equation (3) [24]:

(3) $$TR(u_{i},u_{j},mes)=\frac{\left(\ln Nmes_{j}-1)(\ln(Nmes-Nmes_{j})-1\right)}{\ln(Nmes_{j}•\ln(Nmes-Nmes_{j}))-\frac{\ln(Nmes-Nmes_{j})-\ln Nmes_{j}}{3}}\;TR(u_{i},u_{j},\mathrm{spe})=\alpha •\frac{\left(\ln T{\mathrm{spe}}_{j}-1)(\ln(Tspe-Tspe_{j})-1\right)}{\sqrt{\ln(Tspe_{j}•\ln(Tspe-Tspe_{j}))-\frac{\ln(Tspe-Tspe_{j})-\ln Tspe_{j}}{3}}}\;+\beta •\frac{\left(\ln N{\mathrm{spe}}_{j}-1)(\ln(Nspe-Nspe_{j})-1\right)}{\ln(Nspe_{j}•\ln(Nspe-Nspe_{j}))-\frac{\ln(Nspe-Nspe_{j})-\ln Nspe_{j}}{2}}$$

Here *TR*(*u_i_,u_j_,mes*) is the credibility between users *u_i_* and *u_j_* with the message communication service. *Nmes_j_* is the total number of the messages received by *u_j_* from *u_i_*, *Nmes* is the total number of messages sent by user *u_i_*. *TR*(*u_i_,u_j_,spe*) is the credibility between users *u_i_* and *u_j_* with the speech communication service. *Tspe_j_* is the total conversation frequency received by *u_j_* from *u_i_*, *Tspe* is the total conversation frequency called by user *u_i_*. *Nspe_j_* is the total number of the speech received by *u_j_* from *u_i_*, *Nspe* is the total number of speech called by user *u_i_*.

When the communication duration or frequency between the mobile users increases, the trust growth will drop, which conforms to the diminishing effect theory. This paper uses the logarithmic function to measure the influence on trust degree caused by the speech duration, speech frequency and the along duration [17]. In addition, the trust between mobile users will change with time, because the credibility of intimate university friends decreases with the reduced contact after graduation. Then, this paper adopts the forgetting curve [25] as attenuation function to map the trust degree among mobile users. The specific time attenuation function is shown in Equation (4) [24].

(4) $$f(t_{k},t_{m})=\{\begin{array}{c} \frac{10}{1+e^{\lambda (t_{k}-t_{m})}}\begin{array}{cc} & t_{k}\neq t_{m} \end{array}\\ \begin{array}{cc} 1 & \begin{array}{cc} & \begin{array}{cc} & \begin{array}{cc} & t_{k}=t_{m} \end{array} \end{array} \end{array} \end{array} \end{array}$$

Here the parameter *k* is used to adjust the diminishing rate of the trust degree among mobile users. Equation (5) shows the context-awareness mobile user trust degree based on Equations (3) and (4) [24]:

(5) $$TR{\left(u_{i},u_{j},t\right)}_{c_{k}}=\beta_{1}\sum_{n=1}^{N_{\mathrm{spe}，t}}f(t_{n},k)\ln(\frac{length_{c_{k},u_{i},u_{j},t_{n}}}{length_{c_{k},u_{i},t}}\;+1)+\beta_{2}\sum_{n=1}^{N_{\mathrm{spe}，t}}f(t_{n},k)TR(u_{i},u_{j},spe)+\beta_{3}\sum_{n=1}^{N_{mes，t}}f(t_{n},k)TR(u_{i},u_{j},mes)+\beta_{4}\sum_{n=1}^{N_{\mathrm{dura}，t}}f(t_{n},k)\ln(\frac{duration_{c_{k},u_{i},u_{j},t_{n}}}{duration_{c_{k},u_{i},t}}+1)$$

Here *c_k_* belongs to the context set regulated by Equation (1), *length_ck,ui,uj,tn_* and *duration_ck,ui,uj__,tn_*, respectively, indicate the nth speech duration and the total speech duration between users *u_i_* and *u_j_*. *length_ck,ui,,t_* and *duration_ck,ui,t_* respectively indicate the total speech duration of user *u_i_* and the total along duration of user *u_i_* with all the other mobile users constrained by context *c_k_* before time *t.* *N_spe,t_*, *N_mes,t_* and *N_dura,t_* respectively indicate the speech frequency, message frequency between users *u_i_* and *u_j_* and total along frequency of user *u_i_* with all the other mobile users constrained by context *c_k_* before time *t*, and *t_n_* indicates the time of the nth communication. Since the influences caused by different factors are not even, here weight *β* is used to adjust the influences of different factors, and $\sum_{i=1}^{4}\beta_{i}=1$.

The influences on the trust degree of the same mobile user’s behavior vary with different contexts. For example, with the same acquaintance time, compared with colleagues at work, the target users tend to have more credibility with object-friends at the party. Therefore, when we calculate the trust degree between users, in addition to the time spent together, the corresponding location information should also be considered, as shown in Equation (6) [24]:

(6) $$TR(u_{i},u_{j},t)=\sum_{k=1}^{\mathrm{CN}}\alpha_{k}\times TR{\left(u_{i},u_{j},t\right)}_{c_{k}}$$

Here *CN* indicates the total number of the context classification which can be calculated by the context instances. In this paper, there are 12 (2 × 6 = 12) kinds of different time contexts and 4 kinds of different location contexts, so the total number of context instances of CN is 48 (12 × 4 = 48). *α_k_* ∈ (0,1) represents weighting parameter, used to adjust the influence degree of each context category to trust. The particle swarm optimization algorithm is used to select the most optimal weighting parameter values.

In addition, credibility has transitivity. For example, if users *u_i_* and user *u_l_* do not have direct correspondence, but there is a correspondence between *u_i_*, *u_j_* and *u_j_, u_l_*, then there exists a trust degree between *u_i_* and *u_l_*. Since the trust propagation tends to attenuate, a method is put forward to calculate the trust propagation distance, according to the six-degree segmentation theory and the method provided by [26], as shown in Equation (7) [24]:

(7) $$\mathrm{DIS}=\{\begin{array}{ll} \left\lfloor \frac{\ln n}{\ln k}\right\rfloor & \frac{\ln n}{\ln k}<6\\ 6 & \frac{\ln n}{\ln k}>6 \end{array}$$

where: $k=\frac{\sum_{i=1}^{n}G_{i}}{n}$.

Then, based on Equations (6) and (7), a more accurate context awareness trust degree can be obtained by Equation (8). Here *R* is the effective path to connect mobile user *u_i_* and mobile user *u_j_* [24].

(8) $$T(u_{i},u_{j},t)=\{\begin{array}{c} TR(u_{i},u_{j},t)\begin{array}{ccc} & & L=1 \end{array}\\ \frac{\sum_{\left(u_{i},u_{k},...,u_{m},u_{j}\in R\right)}TR(u_{i},u_{k},t)\times TR(u_{k},u_{l},t)\times ...\times TR(u_{m},u_{j},t)}{L\times \sum_{D(u_{i},u_{k})=1}TR(u_{i},u_{k},t)}\begin{array}{cc} & 1<D(u_{i}.u_{j})\leq L \end{array}\\ 0\begin{array}{cccc} & & & \begin{array}{cccc} & & & \begin{array}{cccc} & & & \min(D(u_{i}.u_{j}))>L \end{array} \end{array} \end{array} \end{array}$$

#### 3.2.5. Interest Similarity-Based Neighbor Finding Approach

It is of one-sided, if only trust degree between different users is used to select the approximate neighbors. For example, if user *u_i_* uses 20 kinds of mobile web services, user *u_j_* uses 25 kinds of mobile web services, but there are only two common mobile web services for both of them, then even though users *u_i_* and *u_j_* have frequent communication behavior, they cannot be considered as approximate neighbors.

Therefore, this paper calculates the preference similarity degree between different users with the evaluation score about a certain mobile web service provided by a mobile user. An improved Pearson correlation coefficient [27] is adopted to calculate the preference similarity degree between different mobile users, as shown in Equation (9) [24]:

(9) $$\{\begin{array}{c} \mathrm{InterSim}（u_{i},u_{j},t)=\frac{\sum_{s\in CI(u_{i},u_{j},t)}\left(SI_{u_{i}s,s}-AverS_{u_{i},t})(SI_{u_{j},s,s}-AverS_{u_{j},t}\right)}{\sqrt{\sum_{s\in CI(u_{i},u_{j},t)}{\left(SI_{u_{i}s,s}-AverS_{u_{i},t}\right)}^{3}{\left(SI_{u_{j},s,s}-AverS_{u_{j},t}\right)}^{3}}}\begin{array}{cc} & {|\mathrm{CI}(u}_{i},u_{j},t)|>=\delta •\min(|I_{u_{i}},t|,|I_{u_{j}},t|) \end{array}\\ \begin{array}{ccc} & & \begin{array}{cc} \begin{array}{ccc} \begin{array}{ccc} \begin{array}{ccc} \begin{array}{ccc} \begin{array}{ccc} 0 & & \end{array} & & \end{array} & & \end{array} & & \end{array} & & \end{array} & {|\mathrm{CI}(u}_{i},u_{j},t)|<\delta •\min(|I_{u_{i}},t|,|I_{u_{j}},t|) \end{array} \end{array} \end{array}$$

Here, *InterSim*(*u_i_*,*u_j_*,*t*) is the interest similarity score between users *u_i_* and *u_j_* at time *t*, *CI*(*u_i_,u_j_,t*) is the common interest web services between users *u_i_* and *u_j_* at time *t*, *SI_ui,s,t_* is the interest score given by user *u_i_* for web service *s* at time *t*, *SI_uj,s,t_* is the interest score given by user *u_j_* for web service *s* at time *t*, *AverS_ui,t_* is the average interest score of all the web services used by user *u_i_* at time *t*, *AverS_uj,t_* is the average interest score of all the web services used by user *u_j_* at time *t*, |*CI*(*u_i_,u_j_,t*)| is the number of the common interest web services between users *u_i_* and *u_j_* at time *t*, |*I_ui_,t*| is the number of the interest web services of user *u_i_* at time *t,* |*I_uj_,t*| is the number of the interest web services of user *u_j_* at time *t*, and *δ* is a threshold used to calculate the minimum number of interest web services between users *u_i_* and *u_j_* at time *t*.

#### 3.2.6. Time Attenuation Function Based Neighbor Finding Agent

Since user preference will present a tendency to decay over time, this paper includes the trust degree, similarity degree calculated by Equations (8) and (9) and time attenuation function to find approximate neighbors with similar interests.

This paper chooses a logistic function to calculate the time attenuation based user preference weight, as shown in Equation (10) [24]:

(10) $$W(u_{i},u_{j},t_{m})=\{\begin{array}{c} \sum_{k=0}^{m}f(t_{k},t_{m})•(\phi •InterSim(u_{i},u_{j},t_{m})+\eta •TR(u_{i},u_{j},t_{m}))\begin{array}{cc} & TR(u_{i},u_{j},t_{m})>=\omega \end{array}\\ \sum_{k=0}^{m}f(t_{k},t_{m})•(\phi •\frac{InterSim(u_{i},u_{j},t_{m})}{\min((AverS_{u_{i},t}),(AverS_{u_{j},t}))}+\eta •\frac{TR(u_{i},u_{j},t_{m})}{N})\begin{array}{cc} & TR(u_{i},u_{j},t_{m})<\omega \end{array} \end{array}$$

Here the parameter *λ* is used to adjust the attenuation of the trust degree between different mobile users, *ϕ* and *η* are the harmonic weighting parameters between 0 and 1, *ω* is the trust threshold between 0 and 1. Finally, the users with higher weight than the average are chosen as the neighbors of the user *u_i_.*

## 4. Simulation, Results and Discussion

### 4.1. Simulation Data Sets

The experiment uses the MIT Reality Mining dataset. It is an open dataset that contains contextual information and the behavior of mobile users using different kinds of mobile network services. It includes a total of 181,797 voice records and text messages of 94 mobile users collected from September 2004 to July 2005. Since the communication row records of mobile users 23, 75, 102, 105 are empty, their data are not adopted in this paper, hence we have a total of 90 valid mobile users. The dataset also includes 94 friendship relations between the mobile users obtained by questionnaire. However, due to the device delays and some noise data such as spam messages and harassing phone calls, the data need pre-processing before the experiment. Then, the data are to be pre-processed and the physical location needs to be deduced, such as at home, at work, or at other places derived by setting up the corresponding rules. Finally, 130 friends’ relationship records are acquired through data analysis as the standard to evaluate the accuracy of trust.

In addition, the data set contains 203 kinds of mobile phone users’ behavior. In this experiment, the context information includes time and location information. Because of the small amount of data in the mobile network service MIT Reality Mining dataset, and few types of context, the simulation data set Mobile Services are used to constrain the context rules of mobile users’ historical behavior and behavior change. The simulated data covers 500 mobile users’ behaviors over six consecutive months including 100 mobile Internet services. The context information of this study includes time and position.

### 4.2. Simulation Steps

**Figure 4:**
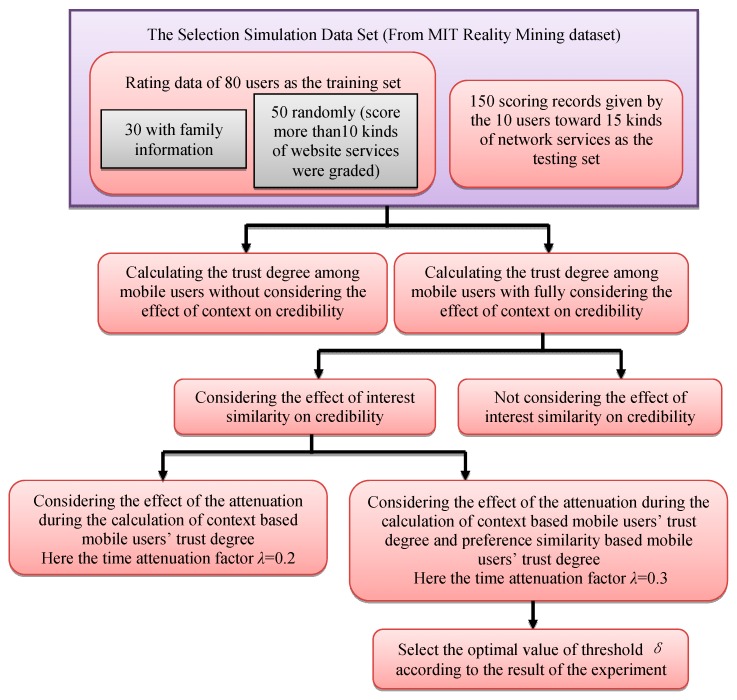
The flow diagram of the simulation steps.

In order to evaluate the accuracy of the neighborhood search, it is necessary to develop a test client displaying the results of the search. We constructed an HDFS Cluster to simulate the proposed method (a single computer with an Intel Core i7 CPU, 4 GB RAM with Windows 8 as NameNode, and two computers with Intel Core i7 CPUs and 4 GB RAM with Windows 8 as DateNode). Java, Matlab 2015 is used as the programming language and the Eclipse integrated development environment. The flow diagram of the simulation steps is shown in Figure 4:

*Step 1*: Select data of the first five months as a training set and those of the sixth months as testing set. This experiment selects rating data of 80 users as the training set. Selection rules are as in the following: firstly, select 30 users with family information, and then select 50 users randomly from network services users scoring more than10 kinds of website services. Test sets are 150 scoring records given by the 10 users toward 15 kinds of network services. In the experiment, the initial credibility among family members is set 1, and that from different families is set 0.
*Step 2*: A logarithmic function is used to analyze the effect of credibility caused by speech duration, speech frequency, message frequency, along duration and along frequency in the trust degree computation, and the context information (time, location and the people around) is taken into consideration. Two approaches are used in the experiment: Equation (5) is used to calculate the trust degree among mobile users without considering the effect of context on credibility, and Equation (8) is adopted when fully considering the effect of context on credibility.

*Step 3*: Add interest similarity to the calculation of credibility with two contrast methods depending on whether the effect of interest similarity on credibility is considered.
*Step 4*: When calculating the trust degree, consider credibility decay and user preference decay with two contrast methods: (1) Consider the attenuation of credibility during the calculation based on context-based mobile users’ behavior. Set time attenuation factor *λ* = 0.2 (here a month is the measure of time) through many experiments, *i.e*., when two mobile users lose contact for more than a year, its credibility decreases to 0; (2) Consider the effect of time context on the credibility calculation based either on mobile users’ behavior or on interest similarity. Through data analysis, this paper sets the preference of the attenuation factor *λ* = 0.3. When *t_k_* − *t_m_* > 18 months, *f* (*t_k,_t_m_*) > 0.045, the user preference decays to 4.5% of the preference in 18 months ago. It can be seen that preference of attenuation speed is slower than that of confidence, because the relationship established between people is longer than that established between people and things.
*Step 5*: Since mobile users can reuse some mobile network services, more repetitions result in greater stability of the mobile user preferences, hence higher credibility. Threshold *δ* = 0.3, 0.4, 0.5 and then select the optimal value according to the result of the experiment.

### 4.3. Results and Discussion

#### 4.3.1. The Effect of Context on the Credibility Among Mobile Users

The weighting parameter values of speech and message interaction are studied by the genetic algorithm as shown in Table 2, and the weighting parameter values of the context including time and location are shown in Table 3. In the above experiment, the attenuation factor of time is not considered, means *λ* = 0, *t_0_* = ∞.

Table 2 shows that the communication behavior among the mobile users plays a more important role in trust degree calculation than the behaviors, and among the behaviours, voice communication behavior has the biggest influence on credibility, because normally the mobile users interact with the familiar people such as friends, families or colleagues by speech communication. The along duration and along frequency is calculated by the two users when they stay within a short distance (such as in the same office), but the persons who stay closely with the mobile users may not be their friends. For example, when the two colleagues in one office are not friends, the along duration and along frequency play a less important role in the calculation of trust degree.

From Table 3 we can see the weighted value of the weekend is greater than that on workdays in most cases and the weighted value at work (morning and afternoon) is less than that of other time periods, because the users normally spend weekends with their families and friends, while they spend weekdays with their colleagues. Therefore, the mobile users’ behaviors in weekends are more important in the calculation of trust degree. Furthermore, the weights of the context in working hours (morning and afternoon) are lower than other time, because the mobile users normally stay with their colleagues at working hours, while they stay with their families or friends at noon or evening. Besides, the weights of the context at home or party are highest than other places, and those of working places are the lowest, because the mobile users stay with their families at home, while they stay with their friends at parties or places such as at theatres and restaurants.

**Table 2:** The weighting parameter values studied by the genetic algorithm.

*ᵝ_1_*	*ᵝ_2_*	*ᵝ_3_*	*ᵝ_4_*
0.5132	0.3263	0.1968	0.1039

**Table 3:** The context weighting parameter values studied by the genetic algorithm.

Context	Weighting Parameter	Context	Weighting Parameter	Context	Weighting Parameter	Context	Weighting Parameter
**000(*ᵃ_1_*)**	0.0028	001(*ᵃ_9_*)	0.9124	002(*ᵃ_17_*)	0.0416	003(*ᵃ_25_*)	0.8636
**010(*ᵃ_2_*)**	0	011(*ᵃ_10_*)	0	012(*ᵃ_18_*)	0.0337	013(*ᵃ_26_*)	0
**020(*ᵃ_3_*)**	0.2316	021(*ᵃ_11_*)	0	022(*ᵃ_1_*_9_)	0.2739	023(*ᵃ_27_*)	0
**030(*ᵃ_4_*)**	0.1372	031(*ᵃ_12_*)	0	032(*ᵃ_20_*)	0	033(*ᵃ_28_*)	0
**100(*ᵃ_5_*)**	0.5798	101(*ᵃ_13_*)	0	102(*ᵃ_21_*)	0.2518	103(*ᵃ_29_*)	0
**110(*ᵃ_6_*)**	0.3216	111(*ᵃ_14_*)	0.0409	112(*ᵃ_22_*)	0	113(*ᵃ_30_*)	0.0357
**120(*ᵃ_7_*)**	0.0835	121(*ᵃ_15_*)	0.6031	122(*ᵃ_23_*)	0.0971	123(*ᵃ_31_*)	0.5894
**130(*ᵃ_8_*)**	1	131(*ᵃ_16_*)	0.9981	132(*ᵃ_24_*)	0	133(*ᵃ_32_*)	0.8873

The above results reveal obvious influence of context factors on trust among users, so when calculating the credibility between mobile users, the context information needs to be considered at the time when the mobile user behavior happens.

#### 4.3.2. The Effect of Time Decaying Factor *λ*

*Precision Ratio*, *Recall Ratio*, and *Mean Absolute Error* are used to analyze the effect of the time decaying factor λ and trust degree threshold. The *Precision Ratio* is the proportion of retrieved documents relevant, defined as Equation (11). The *Recall Ratio* is the proportion of relevant documents retrieved, defined as Equation (12). The *Mean Absolute Error* Here (*MAE*) is the common index used to evaluate the mean absolute error between mobile user preference through study the context and the real context-based user preference, defined as Equation (13):

(11) $$PrecisionRatio=\frac{\#relevantitemsretrieved}{\#retrieved\begin{array}{c} items \end{array}}=P(relevant|retrieved)$$

(12) $$RecallRatio=\frac{\#relevantitemsretrieved}{\#relevant\begin{array}{c} items \end{array}}=P(retrieved|relevant)$$

(13) $$MAE=\frac{1}{n}\sum_{i-1}^{n}|p_{i}-{\overset{\wedge }{p}}_{i}|$$

Here *p_i_* indicates the real user preference based on the context, $\overset{\wedge }{p_{i}}$ indicates the user preference through studying the context.

**Figure 5:**
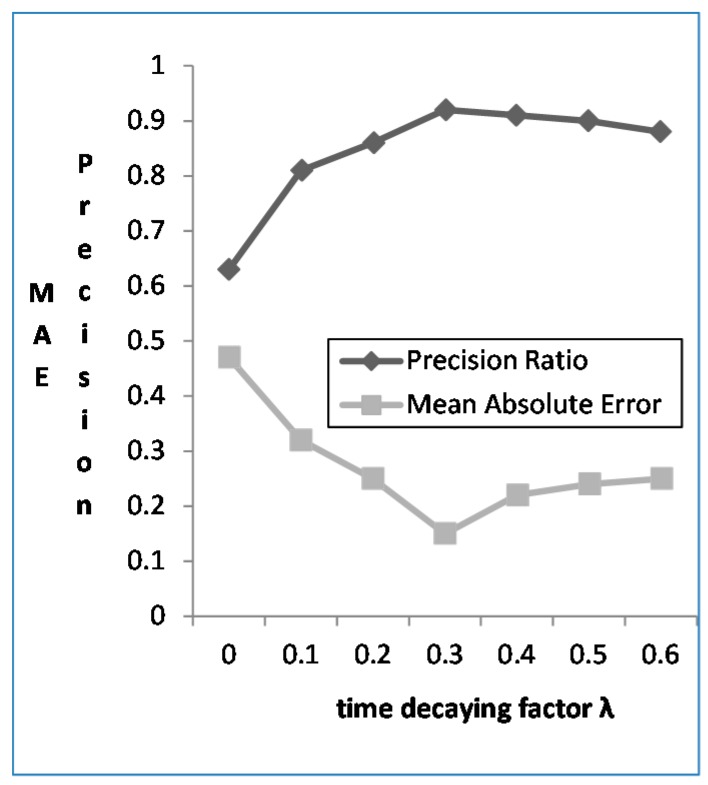
The *Precision* and *MAE* based on the different value of *λ*.

From the Figure 5 we can see that the mobile user preference weights studied by context are good when considering the time decaying factor (*λ* = 0.3), with 18.97% increase for the *Precision Ratio*. In contrast, when not considering the time decaying factor (*λ* = 0), the *MAE* is decreased by 31.26%. When the *λ* > 0.3, the *Precision Ratio* declines, because the context-based user preference would decay over time, and the decay rate of the mobile users depends on the time decaying factor. The decay rate is slow when the *λ* is small, and the accuracy of the user preference through study is not high; while the decay rate is fast when the *λ* is big, but the accuracy of the user preference through study is still not high.

#### 4.3.3. The Effect of Trust Degree Threshold

**Figure 6:**
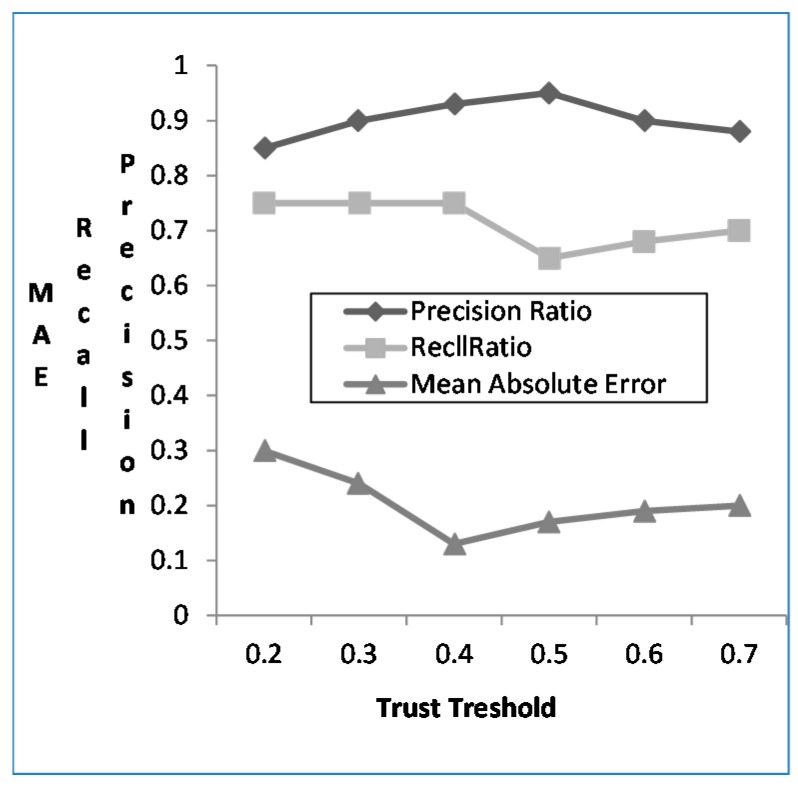
*Recall* and *Precision* for different trust threshold.

With the increase of trust degree threshold, the prediction accuracy of the mobile user preference is improved. When the threshold *δ* = 0.5, the precision is the highest, and when threshold *δ* ≤ 0.4, the recall rate remains the same. When the threshold *δ* > 0.4, the recall rate declines. By combining all the analysis results, it reveals that when reliability threshold is large, even if the precision in predicting user preference is improved, the corresponding recall is declining, as shown in Figure 6. Therefore, in order to guarantee the recall, the threshold *δ* cannot be too large. This paper selects the trust threshold *δ* as 0.4.

Table 4 shows the precision and the number of the correct nodes when considering interest similarity and when not considering it.

**Table 4:** Results in different classification methods.

Classification Method	Precision	The Number of the Correct Nodes
Not considering interest similarity	0.62	35
Considering interest similarity	0.73	41

### 4.4. The Comparative Result of the Proposed Method and the Other Two Methods

**Figure 7:**
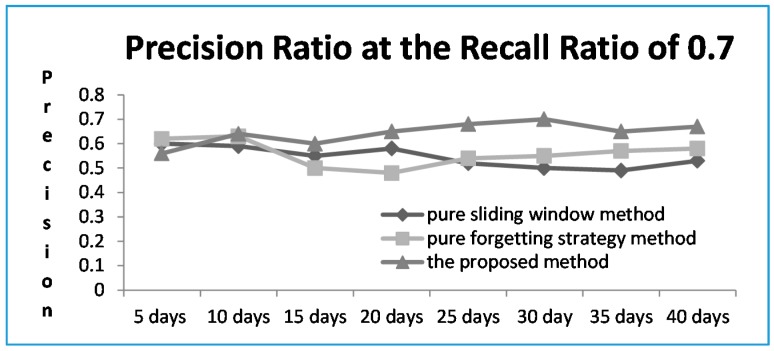
The precision ratio of the proposed method at the recall ratio of 0.7.

By combining all the analysis results, when the reliability threshold is large, even if the precision in predicting user preference is higher, the corresponding recall is declining. This paper therefore selects the trust threshold *δ* as 0.4. Based on the selected threshold, the results are compared with the other two methods: one is the pure sliding window method which can grasp the user’s short-term performance more accurately based on the recent data collected from desktop as reference, and the other one is the pure forgetting strategy method which mainly considers users’ and their neighbors’ previous interest factor. Figure 7 shows the precision ratio at the recall ratio of 0.7.

It can be seen from the simulation result that for the pure sliding window method, when the user’s short-term behavior is outstanding, the performance is better than that of the pure forgetting strategy method, but the overall performance of the pure forgetting strategy method is better than that of the pure sliding window method because the pure forgetting strategy method considers users’ and their neighbors’ previous interest factor. The proposed method comprehensively considers the trust degree and relevance degree for different services, and the neighbors are updating constantly according to users’ changing interests; therefore, our proposed method outperforms the other two methods.

## 5. Conclusions

Nowadays, more users are beginning to use mobile networks to retrieve information, and as such, mobile users with similar preferences can determine the choice of the neighbors with the approximate preferences to a large extent. The present research therefore introduces the trust degree, similarity degree and the time attenuation trend between users to calculate the mobile user preference similarity, on the basis of which, it can update the target users according to the dynamic personalized preferences set up by neighbor users with similar preferences.

First, the direct and indirect trust degree between users are calculated based on users’ speech duration, speech and message frequencies. Then, the improved Pearson Correlation Coefficient method is adopted to calculate the preference similarity degree between different users based on the evaluation score about a certain mobile web service given by a mobile user. Finally, the trust degree, similarity degree and time attenuation function are comprehensively used to find the potential approximate neighbors with similar interests so as to update the target user’s preference profile according to the neighbors’ preference profile.

The proposed method is compared with the pure sliding window method and the pure forgetting strategy method. The results show that the proposed approach outperforms the other two approaches in terms of *Recall Ratio* and *Precision Ratio*. However, this research has two limitations. Firstly, in order to capture the context-aware user preference, the current method has to analyze the behavior of mobile users, which brings about a privacy protection problem. Secondly, the current method is based on the independent or simplified relevance of context, so how to specify the relevance of context more precisely needs to be improved.
