# Correction: Gao, Q. et al. A Context-Aware Mobile User Behavior-Based Neighbor Finding Approach for Preference Profile Construction. *Sensors* 2016, *16*, 143

**DOI:** 10.3390/s16081230

**Published:** 2016-08-04

**Authors:** Qian Gao, Deqian Fu, Xiangjun Dong

**Affiliations:** 1 School of Information, Qilu University of Technology, #3501 Daxue Road, Changqing District, Jinan 250353, China; dxj@qlu.edu.cn; 2 School of Informatics, Linyi University, Shuangling Rd., Lanshan, Linyi 276005, China; fudeqian@lyu.edu.cn

At first, may I offer my profoundest respects to the previous work obtained by the author Shi, whose work enlarged our view and set a good research direction for us and enlightened the initial idea of our paper. Second, when we borrowed the idea of the trust degree calculation and some equations proposed by Shi [1], we did not make a proper attribution, for which we sincerely apologize and we have great respect and are making a full acknowledgment of the previous authors’ work. However, we improved the trust degree calculation method as the influence factor to find mobile users with similar interests in our paper, based on which we updated the user preference of the target user by the neighbor users’ preference profiles.

The ultimate goal of our research is to monitor humans’ activities and the integration between humans and agents so as to build and update the Personalized Ontology Profile based User Preference Profiles, based on which we provide the users with more accurate retrieval results. The existing methods mainly focused on the analysis of Internet users’ behavior-related context information or mobile user related context information (time, location) separately, without analyzing both kinds of context information comprehensively, hence exhibiting unilateralism.

In order to solve this problem, this paper firstly innovatively proposed an idea to comprehensively consider three kinds of context information to update the Personalized Ontology Profile based User Preference Profiles. First we made use of the local device-related context information (such as documents, E-mail, pictures and so on) to construct the Personalized Ontology Profile (POP) by considering the domain ontology, knowledge structure, and document information stored on all of the smart devices that belong to the same user; then, based on Internet users’ behavior-related context information (interactive historical information and user information related with the retrieval), we constructed a user preference profile which can comprehensively track the users’ long-term behaviors and short-term behaviors by using two kinds of different user interest tracking strategies (Sliding-Time-Window Update Strategy for short-term preferences, and Time-Based-Forgetting Function Update Strategy for long-term preferences). Finally we make use of the mobile network users’ behavior-related context information to find the mobile users with frequent and effective voice interactions, message interactions, meaningful position contexts and many different shared mobile services so as to update the user preference of the target user by the neighbor users’ preference profiles.

Second, there are more messages and voice interactions between mobile users between 5:30 p.m. and 9:30 p.m. on weekdays between colleagues, while the interactions are more often between friends or families on the weekend. Furthermore, the people in the party who are within a close distance always show a high trust degree, so we further refined the clarification of the mobile user-related context proposed by Shi [1], newly increasing two kinds of time contexts and one kind of position context so as to make the context information more accurately reflect the influence of different kinds of time and position contexts on users. The effectiveness of the three types of newly added context information can be seen in the simulation part. However in this part, we failed to add a reference to the work by Shi to explicate our improvement on the classification of the context proposed by Shi.

Third, the communication frequency and times can highly reflect the credibility of the two mobile users, and the mobile users are more inclined to send text message using social software instead of the mobile phone, so this paper especially weakened the effects of the text message by only considering the total number of messages received by u_j_ from u_i_ and the total number of messages sent by user u_i_. Furthermore, because the speech communication frequency act on the calculation of the users’ trust degree is more effective than the number of times of the speech communication act on the calculation of the users’ trust degree, this paper innovatively proposed two different approaches to calculate the credibility degree caused by frequency and times, and further set the weights *α* and *β* to adjust the influences of different factors (see Equation (3)).

Fourth, we found that the traditional forgetting curve drops too fast. For friends, if they are apart for months or years, the speech call frequency between them may decline, but they still contact each other through many other means, such as Twitter or other chatting software. Therefore, on the basis of the idea of using, for reference, the logarithmic function to measure the influence on the trust degree caused by the speech duration, speech frequency and the along duration [1], this paper further improved the forgetting curve to measure the attenuation of time (see Equation (4)), and innovatively comprehensively considered the credibility degree calculated by Equation (3) and the context-constrained duration time to obtain more accurate and effective context-awareness mobile user trust degree. Furthermore, in the first line beneath Equation (4), the name of the parameter k should be changed from k to λ, and all of the f(t_n_, k) in Equation (5) should be changed to f(t_k_,t_n_).

Fifth, we adopted the method proposed by Shi [1] and the six-degree segmentation theory [2] to further calculate the context awareness trust degree between mobile users by further considering the corresponding location information and the indirect trust propagation. Then we adopted the idea of the preference similarity degree calculation [1] to measure the relationship between two users by the evaluation score of a certain mobile web service. However in this part, we failed to add a reference to the work by Shi when we used her idea. Moreover, because the ultimate goal of our research is to find users with close relations, so as to update the weight of each term in the ontology-based user preference profile, the common mobile web services used by two users are much more important than the total mobile web services used by each user. Based on this finding, we made a slight improvement, raised the effect of the number of common mobile web services, and improved the exponent from 2 to 3. Furthermore, the name of the trust propagation distance in Equation (8) should be changed from L do DIS.

Finally, a novel context-awareness mobile user preference neighborhood-finding agent is used to identify the neighbors with similar interests by comprehensively considering the time attenuation, trust degree and similarity degree (see Equation (10)). The users with a higher weight than the average are chosen as the neighbors of user u_i_, based on which the terms in the neighborhood’s preference profile will be added as a new interest into the user’s preference profile. This part is the new proposed idea, which can further increase the accuracy of the findings of the approximate neighbors.

The simulation in this paper is divided into two parts:

The first part is to test the effects of all the improvement and the enhancement proposed in this paper. We borrowed the simulation strategy of Shi [1] to test the proposed improved new approach. Since one of the important improvements in this paper is the refinement of the context clarification, we first simulated and obtained the weights of different kinds of contexts. From the result we can see that the influence of the mobile users’ behaviors during weekends from 5:30 p.m.to 9:30 p.m. plays a more important role than those during the workday from 5:30 p.m.to 9:30 p.m. Furthermore, the weights of the context of being at home or at a party are higher than those in other places, and those of working places are lower, because the mobile users stay with their families at home while they stay with their friends at parties or in places such as at theaters and restaurants, as shown in Table 3. Hence, the results show that the first improvement is effective. Then, based on the refined context clarification and improved trust degree calculation methods (shown in dialogues 5 and 6 in this correction), we achieved different effects of the time decaying factor and trust degree threshold.

The second part is to test the precision ratio of the personalized retrieval based on the updated user preference profile according to the neighborhood’s user preference profile. We kept testing it with the platform of the PMY (Personalized Multiagent Retrieval) System with two other methods for more than one month. The simulation results show that our proposed method outperforms the other two methods.

## References

[1] Yan C.S. (2013). Research on Dynamic Acquisition of Context Mobile User Telecommunication
Data. Ph.D. Thesis.

[2] Zhang S., Zhong H. (2013). Trust Network and Small World Trust Community Clustering for
E-Commerce. Int. J. Hybrid Inf. Technol..

